# Genome editing: from tools to biological insights

**DOI:** 10.1186/s13059-018-1570-6

**Published:** 2018-11-06

**Authors:** Yixin Yao

**Affiliations:** Genome Biology, BMC, Shanghai, 200040 China

DNA cleavage technologies, such as zinc-finger nucleases (ZFNs), transcription activator-like effector nucleases (TALENs), and clustered regulatory interspaced short palindromic repeat (CRISPR)/Cas-based DNA/RNA endonucleases, have proven to be powerful tools. The genome editing tool box is continually expanding its dimensions to higher precision, larger scale, and diversified applications to enable us with exciting biological insights that were not available to us before [1]. The past few years have witnessed the heated debate of genome engineering safety, as the technique is broadly applicable to agricultural species as our food source, eventually humans and our cells as well. Yet despite the debate, the field continues to expand with the creation of better tools, and each answer seems to inspire more questions. We are pleased to invite you to read the first articles in our special issue entitled “Insights from Genome Editing”, which features the advances in the field and the insights gained from application of genome editing tools.

Although many CRISPR-associated proteins have been applied to genome editing and shown potential for gene therapy, less than a handful have been demonstrated to work as a part of in vivo editing platforms. With its smaller size and distinct protospacer adjacent motif (PAM), *Neisseria meningitidis* Cas9 (NmeCas9) provides a unique advantage. Ibraheim and colleagues [2] demonstrate its gene therapy potential by in vivo delivery of NmeCas9 and its guide in a single expression cassette that is sufficiently small for all-in-one recombinant adeno-associated vectors (rAAV). By targeting *Pcsk9* and *Rosa26* genes in adult mice, they are able to observe efficient editing with high specificity. The immune responses as well as the prolonged expression of NmeCas9 (up to 50 days) in vivo are also investigated to fully understand the therapeutic potential of this platform.

On the other hand, base editors are believed to be more efficient and produce fewer undesirable mutations. In this special issue, Li and colleagues [3] apply and optimize the adenine base editors in crops including rice and wheat. They show that their high efficiency could increase the reliability of genome engineering of herbicide-resistant rice plants harboring the C2186R substitution in OsACC. Base editors have been shown to be useful as an alternative tool to silence genes by introducing stop codons in the gene coding sequence [4]. Gapinske et al. design programmable exon skipping called CRISPR-SKIP, by disrupting the guanosine within the splice acceptor of exons in genomic DNA [5]. They find the disruption of this guanosine can be effectively mutated by converting the complementary cytidine to thymidine using CRISPR-Cas9 C > T single-base editors. The consequences of the programmable exon skipping are complex, which highlights the importance of better understanding of exon–intron architecture and its recognition by the spliceosome machinery.

Despite the variety of the genome editing tools available to us, the concerns of genomic errors introduced by genome editing approaches still remain. Alkan and colleagues [6] bring the binding energy model for the Cas9-gRNA-DNA complex into off-target assessment and find the newly integrated models are in better agreement with experimental results. The findings generated from the nucleic acid duplex energy models indicate the binding energies are one of the new directions of off-target mechanism studies. Guo and colleagues [7] further investigate the behavior of non-homologous end joining in CRISPR-Cas9 mediated genome editing, and find the accuracy of the repair is hindered by frequent + 1 and + 2 insertions in the paired Cas9-gRNA system. A set of rules are summarized to increase precision in out-of-frame and in-frame deletions of defined length by harnessing accurate non-homologous end joining in the system.

The advances in DNA cleavage technologies not only help us to understand the genetic mechanism in gene regulations, but also provide a chance to understand how epigenetic elements affect local and distal regulatory elements in gene transcription. Lei and colleagues [8] review epigenetic editing tools, and how they could regulate transcription without introducing a genetic sequence change, facilitating the research of regulatory elements. Han and colleagues [9] apply a functional CRISPR screen targeting senescence-induced enhancers that are putatively regulated by AP-1 to understand the establishment and maintenance of oncogene-induced senescence. Guo and colleagues [10] use CRISPR as a means to investigate the role of chromatin structure in prostate cancer. Their results reveal that the cancer-related genes could be normally held in a repressive loop by CTCF, and once the CTCF anchor region is weakened by genetic variants, the gene expression could be affected by a distal regulatory mechanism. Finally in a Short Report, Duan and colleagues [11] reveal the importance of in vivo models for imaging of genomic loci. The telomere dynamics in dCas9-EGFP knock-in mouse liver exhibit more constrained anomalous diffusion than that observed in cultured cell lines, which warrants mechanistic study of whether the dynamics of genomic loci are regulated in a tissue-specific and development-specific manner.

More articles from this proceeding field will be published in the coming weeks. Each of the articles from this special issue of *Genome Biology* highlights the opportunities brought about by the advances in biotechnology and how thinking out of the box could bring us new biological insights using tools that already exist.
